# Supplementary material for: Antarctic Rahnella inusitata: A Producer of Cold-Stable β-Galactosidase Enzymes
Source: Int J Mol Sci. 2021 Apr 16;22(8):4144. doi: 10.3390/ijms22084144 (PMC8074230; doi:10.3390/ijms22084144)
Supplement: Supplementary file 1 [file ijms-22-04144-s001.zip › ijms-1106866 sup.pdf]

International Journal of *Molecular Sciences*

MDPI

**SUPLEMMENTARY MATERIAL**

**Table S1.** Antarctic strains with positive detection of β-galactosidase activity based on β-Bluo-Gal reduction detection.

| Strain ID | Nearest taxa (based on 16S rARN BLASTn comparison) | Β-galactosidase activity [a] | Agar diffusion [b] | Max. β-galactosidase activity (U/mL) |
|---|---|---|---|---|
| **34 Sc** | *Arthrobacter sp* | 48 h | | |
| **26Sc** | *Arthrobacter sp* | 48 h | | |
| **5 Sc** | *Arthrobacter cryoconiti* | 48 h | | 182 |
| **36Pc** | N/A | | | |
| **7 Sc** | N/A | 48 h | | |
| **So 9.2** | N/A | | | |
| **22** | N/A | | | |
| **64** | *Arthrobacter sp* | | | |
| **28Pc** | N/A | | | |
| **64 pc** | *Arthrobacter sp* | | | |
| **Cep 68** | N/A | 48 h | | |
| **So 64.3** | *Arthrobacter sp* | 48 h | | |
| **So 64.1** | *Sporosarcina psychrofila* | 72 h | | |
| **So 5b** | *Arthrobacter alpinus* | 72 h | | |
| **So9b** | *Arthrobacter scleromae* | 48 h | | 246 |
| **98** | *Arthrobacter psychrochitiniphilus* | 48 h | | 119 |
| **So 8.10.10** | Non cultivable | 96h | | |
| **63** | *Arthrobacter scleromae* | | | |
| **c vic 80 A** | N/A | | | |
| **Yelcho 5** | *Arthrobacter alpinus* | | | |
| **S 6.10** | N/A | | + | |
| **71** | N/A | | | |
| **31** | N/A | | | |
| **S 11.9** | N/A | | | |
| **Se 8.10.12** | *Rahnella sp.* | 48 h | + | 2157 |
| **Sod3** | N/A | | | |
| **So 30.1** | N/A | | | |
| **C vic 62** | N/A | | | |
| **So 30** | N/A | | | |
| **S 11.3** | *Serratia liquefaciens* | 72 h | | |
| **75 Pc** | *Arthrobacter sulfonivorans* | | | |
| **C vic 28** | N/A | 48 h | | |
| **C vic 15** | *Stenotrophomonas maltophilia* | | | |
| **C vic 82** | *Streptomonas sp.* | 72 h | | |
| **Cp 7.27** | N/A | | | |
| **39 pc** | N/A | | | |
| **10 Scan** | N/A | 48 h | | |
| **C v 71** | N/A | | | |
| **28** | Non cultivable | | | |
| **S1.2** | N/A | | | |
| **So 1c** | N/A | | | |
| **So 5c** | *Arthrobacter sp.* | 96h | | |
| **CSo 30.1** | N/A | | | |
| **11.1 Pc** | *Streptomonas sp.* | | | |
| **1Pc** | N/A | 48 h | | |
| **6 Sca** | N/A | | | |
| **88** | *Arthrobacter sp.* | 48 h | | |
| **8 Sca** | N/A | | | |
| **C vic 68** | N/A | 48 h | | |
| **C vic 85** | N/A | | | |
| **75** | *Arthrobacter sulfonivorans* | | | |
| **49** | N/A | | | |
| **64 pc** | *Arthrobacter sp.* | | | |
| **23 Sc** | N/A | | | |
| **C vic 75** | *Arthrobacter sulfonivorans* | | | |

| | | |
|---|---|---|
| **Cp 4.7** | N/A | |
| **Cp7.12** | N/A | |
| **Cp 50** | N/A | |
| **45** | N/A | |
| **So 30.3** | N/A | |
| **C vic prat** | *Sphingobacterium faecium* | |
| **C vic 12 r** | *Serratia sp* | 72 h |
| **Yelcho 4** | *Arthrobacter Psychrochitiniphilus* | 48 h |
| **11.12** | N/A | 72 h |
| **35 Sc** | *Arthrobacter sp.* | 72h |
| **52 Pc** | N/A | 72h |
| **So 10b** | *Arthrobacter scleromae* | 72h |
| **So 17 1c** | *Stenotrophomonas rhizophila* | |
| **Decet** | N/A | |
| **Se 4.02** | N/A | |
| **C vic 71** | N/A | |
| **So1d** | N/A | |
| **29 sc** | N/A | |
| **65** | N/A | |
| **33 Sc** | *Arthrobacter sp.* | |
| **92** | No cultivable | 72h |
| **So 4.5** | N/A | |
| **58.5 pc** | N/A | 48 h |
| **68** | N/A | 48 h |
| **8 Sc** | N/A | |
| **91** | *Arthrobacter sp.* | |

N/A: No identified strains

a: highest color intensity detected; non-reported strains showed low activity (light blue color)

b: indicative of possible extracellular activity

**Table S2.** Alignment lengths and percentage of identity between nine *Rahnella* species genomes including the Antarctic isolate Se8.10.12. by Average Nucleotide Identity (ANI) calculation using blastn method.

**ANIb Porcentage Identity**

| | R.variigena | R.sp_ERMR1 | R.sp_Y9602 | R.victoriana | R.woolbedingensis | **Antarctic_strain-Se81012** | R.bruchi | R.inusitata | R.aquatilis |
|---|---|---|---|---|---|---|---|---|---|
| R.variigena | 100,000% | 85,151% | 87,967% | 87,824% | 90,925% | **84,390%** | 90,776% | 84,149% | 87,894% |
| R.sp_ERMR1 | 85,191% | 100,000% | 85,172% | 85,464% | 85,011% | **84,691%** | 84,910% | 84,471% | 85,140% |
| R.sp_Y9602 | 87,979% | 85,128% | 100,000% | 88,788% | 87,408% | **84,374%** | 87,181% | 84,067% | 99,575% |
| R.victoriana | 87,850% | 85,395% | 88,794% | 100,000% | 87,211% | **84,683%** | 87,097% | 84,548% | 88,804% |
| R.woolbedingensis | 90,922% | 84,926% | 87,306% | 87,157% | 100,000% | **84,314%** | 93,323% | 84,079% | 87,288% |
| **Antarctic_strain-Se81012** | **84,443%** | **84,732%** | **84,390%** | **84,708%** | **84,438%** | **100,000%** | **84,246%** | **99,047%** | **84,340%** |
| R.bruchi | 90,678% | 84,847% | 87,121% | 86,972% | 93,317% | **84,099%** | 100,000% | 83,889% | 87,068% |
| R.inusitata | 84,156% | 84,473% | 84,077% | 84,510% | 84,134% | **99,042%** | 83,983% | 100,000% | 84,029% |
| R.aquatilis | 87,864% | 85,145% | 99,532% | 88,811% | 87,370% | **84,307%** | 87,109% | 84,034% | 100,000% |

**ANIb Alingment Lengths**

| | R.variigena | R.sp_ERMR1 | R.sp_Y9602 | R.victoriana | R.woolbedingensis | **Antarctic_strain-Se81012** | R.bruchi | R.inusitata | R.aquatilis |
|---|---|---|---|---|---|---|---|---|---|
| R.variigena | 5499108.0 | 3838651.0 | 4181097.0 | 4283792.0 | 4016795.0 | **3559437.0** | 4149954.0 | 3773432.0 | 4165937.0 |
| R.sp_ERMR1 | 3810085.0 | 5530672.0 | 3851166.0 | 3831249.0 | 3770733.0 | **3512016.0** | 3834676.0 | 3700932.0 | 3840761.0 |
| R.sp_Y9602 | 4173062.0 | 3864328.0 | 5614252.0 | 4339729.0 | 3909444.0 | **3589950.0** | 3968411.0 | 3809407.0 | 5247245.0 |
| R.victoriana | 4276676.0 | 3852733.0 | 4345330.0 | 5563295.0 | 3916929.0 | **3558522.0** | 4015161.0 | 3762187.0 | 4342022.0 |
| R.woolbedingensis | 4018336.0 | 3763544.0 | 3928272.0 | 3901674.0 | 5457431.0 | **3526755.0** | 4364029.0 | 3761374.0 | 3912419.0 |
| **Antarctic_strain-Se81012** | **3550583.0** | **3497099.0** | **3576406.0** | **3539610.0** | **3517166.0** | **4704828.0** | **3607730.0** | **4215481.0** | **3536186.0** |
| R.bruchi | 4121409.0 | 3792488.0 | 3950430.0 | 4005238.0 | 4361003.0 | **3596944.0** | 5501702.0 | 3832222.0 | 3943148.0 |
| R.inusitata | 3730309.0 | 3655198.0 | 3786768.0 | 3723719.0 | 3729523.0 | **4194551.0** | 3809462.0 | 4910561.0 | 3772326.0 |
| R.aquatilis | 4151938.0 | 3830309.0 | 5266460.0 | 4340385.0 | 3919960.0 | **3539061.0** | 3971400.0 | 3796544.0 | 5656799.0 |

**Table S3.** Quality evaluation of the three-dimensional models of *Rahnella inusitata* Se8.10.12 β-galactosidases

| Protein | Structure state | QMEAN4 Value | Ramachandran graphic (PROCHECK) | |
|---|---|---|---|---|
| | | | A | B |
| Se.8.10.12 Lac-Z | SWISS-MODEL Output | -1.04 | 1 | 8 |
| | YASARA Output | 0.07 | 1 | 5 |
| Se.8.10.12 BglY | SWISS-MODEL Output | -0.69 | 1 | 4 |
| | YASARA Output | 0.08 | 1 | 3 |
| Se.8.10.12 EbgA | SWISS-MODEL Output | -3.02 | 1 | 10 |
| | YASARA Output | -2.63 | 1 | 7 |

A: Residues in disallowed regions, B: Residues in generously allowed regions

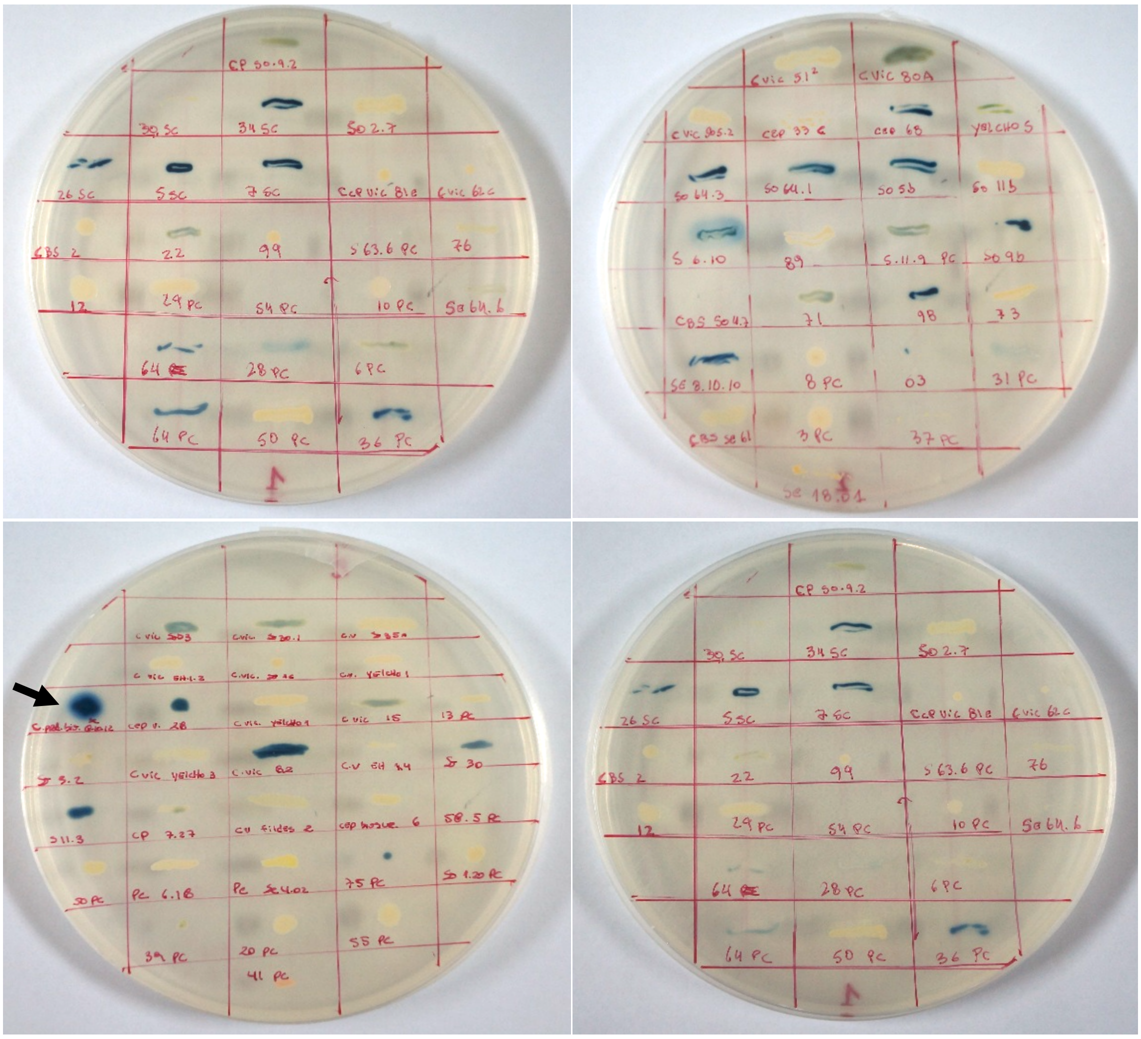

**Figure S1.** Example of β-galactosidase producing Antarctic strains determined in Lactose Agar using β-Bluo-Gal as an indicator of the β-galactosidase activity (blue stripes showing positive activity). Selected strain Se 8.10.12 as promising β-galactosidase enzyme producer is marked with an arrow.

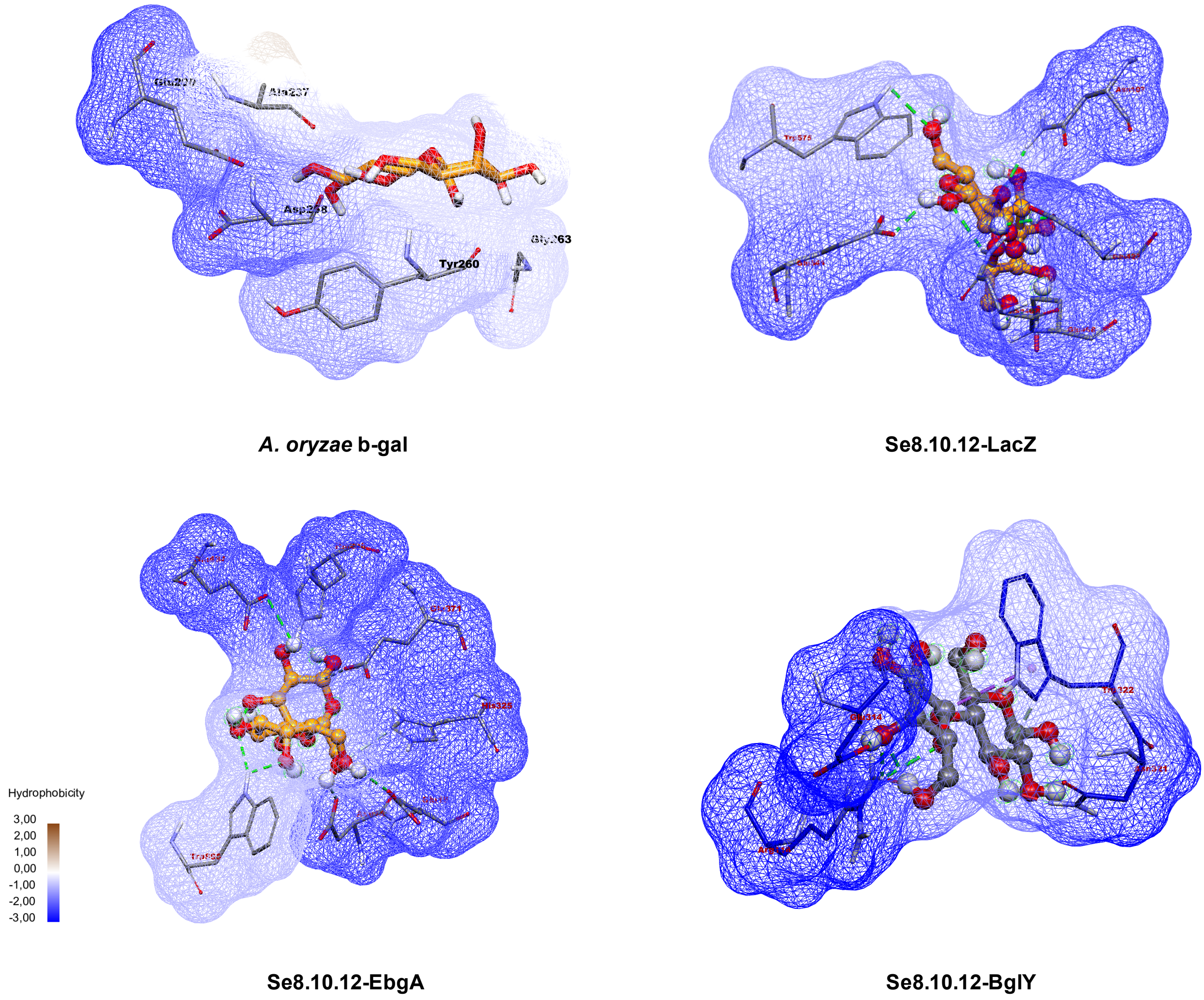


**Figure S2.** The hydrophobicity of the active site in docking analysis of β-galactosidases.
